# Antibodies against SARS-CoV-2 Time Course in Patients and Vaccinated Subjects: An Evaluation of the Harmonization of Two Different Methods

**DOI:** 10.3390/diagnostics11091709

**Published:** 2021-09-18

**Authors:** Ruggero Dittadi, Mara Seguso, Isabella Bertoli, Haleh Afshar, Paolo Carraro

**Affiliations:** Laboratory Medicine Unit, Ospedale dell’Angelo, ULSS 3 Serenissima, Mestre, 30122 Venice, Italy; mara.seguso@aulss3.veneto.it (M.S.); isabella.bertoli@aulss3.veneto.it (I.B.); haleh.afshar@aulss3.veneto.it (H.A.)

**Keywords:** immune response, SARS-CoV-2 antibody response, vaccination, method comparison, harmonization

## Abstract

The time course of antibodies against SARS-CoV-2 is not yet well elucidated, especially in people who underwent a vaccination campaign. In this study, we measured the antibodies anti-S1 and anti-RBD with two different methods, both in patients and in vaccinated subjects. One hundred and eight specimens from 48 patients with COVID-19 (time from the onset of symptoms from 3 to 368 days) and 60 specimens from 20 vaccinated subjects (collected after 14 days from the first dose, 14 days and 3 months after a second dose of Comirnaty) were evaluated. We used an ELISA method that measured IgG against anti-Spike 1, and a chemiluminescence immunoassay that measured IgG anti-RBD. In the patients, the antibodies concentrations tended to decline after a few months, with both the methods, but they persisted relatively high up to nearly a year after the symptoms. In the vaccinated subjects, the antibodies were already detectable after the first dose, but after the booster, they showed a significant increase. However, the decrease was rapid, given that 3 months after the second vaccination, they were reduced to less than a quarter. The conversion of the results into BAU units improves the relationship between the two methods. However, in the vaccinated subjects, there was no evidence of proportional error after the conversion, while in the patients, the difference between the two methods remained significant.

## 1. Introduction

The determination of the antibodies against SARS-CoV-2 could be useful in epidemiological studies, for estimating the spread of the infection and the lethality rate, in the serological diagnosis of individuals with mild or moderate symptoms, and those who are asymptomatic, in the first screening of convalescent patients for plasma collection and in monitoring of the antibody response of vaccinated subjects.

The long-term time course of the antibody response in COVID-19 disease is not yet fully determined. Some studies show a significant decrease in antibody concentrations within 3–4 months from the onset of symptoms [1,2,3]. Other reports find constant or only slightly decreased levels, starting from 4 months and up to 10 months from the symptoms’ onset [4,5,6,7], even when specific neutralizing antibodies [8] were measured. In particular, the time course of the antibody response seems variable, also according to the method used [7,9]. On the other hand, antibodies seem to persist through 4–6 months in vaccinated subjects [10,11].

The aim of this work is to evaluate the performance of two methods for the determination of antibodies to SARS-CoV-2, in patients with a long-term time course and in a little cohort of subjects vaccinated with the Comirnaty vaccine. Moreover, the effectiveness of the transformation of the antibodies concentrations into the international reference standard, for the harmonization of the assays, was evaluated.

## 2. Materials and Methods

We recruited symptomatic subjects who presented at the dell’Angelo Hospital (Mestre, Italy) in March 2020, affected by COVID-19 according to both clinical and laboratory criteria. Forty-eight patients with known date of symptoms’ onset were included in the study (41 males, 7 females, median age 62.3 years, minimum 28, maximum 87). The median time from the onset of symptoms was 26 days (minimum 3, maximum 368). A total of 108 blood specimens were collected. Number of specimens per patient and other patients’ characteristics are reported in Table 1.

Moreover, serum samples were collected from 20 healthcare workers after 14 days from the first dose, 14 days and 3 months after a second dose of Comirnaty vaccine (BNT162b2, BioNTech/Pfizer, Mainz, Germany/New York, NY, USA). All subjects underwent periodical nasopharyngeal swab testing (every 2 or 3 weeks) and had a negative result to the antibody determination prior to vaccine administration.

The specimens were collected in polyethylene tubes (BD Vacutainer^®^; Becton Dickinson, CA, USA) containing clot activator and gel separator, and were centrifuged at 1500× *g* for 10 min and the sera were stored at −80 °C until the assay. IgG were measured with an ELISA method, the “anti-SARS-CoV-2 QuantiVac ELISA IgG” (Euroimmun, Lubeck, Germany) and a two-step chemiluminescence microparticle immunoassays for the determination of IgG against RBD (receptor binding domain) “SARS-CoV-2 IgG anti-RBD” (SNIBE, Shenzhen, China).

Both the assays were performed according to the manufacturer’s instructions. Briefly, in the ELISA Euroimmun method 100 µL of the calibrators, controls or diluted samples were added per well and incubated for 1 h at 37 °C. After washing, in each well 100 µL of enzyme conjugate and, after incubation for 30 min at 37 °C, 100 µL of chromogen/substrate solution were added. The plate was incubated for 30 min at room temperature, the reaction was stopped and the color reaction was read at 450 nm. The concentrations of the antibodies against the S1 protein were determined through the interpolation with a six-point calibration curve (from 1 to 120 relative units/mL). Results < 8 RU/mL were considered as negative, results > 8 RU/mL and ≤ 11 RU/mL were considered as indeterminate and >11 RU/mL aPlease verify changes to this titles positive. 

The IgG anti-RBD method measures antibodies against receptor binding domain of the S1 protein and was carried out on the analyser Maglumi 800 (SNIBE, Shenzen, China). A nine-point master curve was used (from 1 to 100 arbitrary units/mL), periodically adjusted by a 2-point calibration. Results ≥ 1 AU/mL were considered as positive.

The good analytical characteristics of the two methods and the satisfactory correlation with the neutralization tests were previously evaluated and confirmed [12,13,14]. The concentrations were measured taking into consideration the previously determined reportable range of the respective methods [15].

The respective cut-offs were determined by the manufacturers on the basis of a specificity of 99.8%, and confirmed by independent studies [13,14].

The units of the respective assays were established by the manufactures and are specific for each method.

An international reference serum standard for SARS-CoV-2 antibody testing, expressed as binding antibodies units, has been developed by the National Institute for Biological Standards and Control (NIBSC) [16]. The reference standard (WHO 20/136) has been used to calibrate antibody testing systems against an international reference protocol. Correction factors versus the international standard were determined for both assays (4.33 for Maglumi and 3.2 for ELISA).

The statistical analyses were performed with MedCalc © Software, version 7.4.2.0 (MedCalc Software, Mariakerke, Belgium).

## 3. Results

The overall concordance rate between the methods was 89.8% (kappa statistics, 0.54; 95% CI, 0.30–0.78). 

The results of the patients’ specimens were subdivided into seven groups, for which the qualitative performance was evaluated (Table 2).

**Figure 1:**
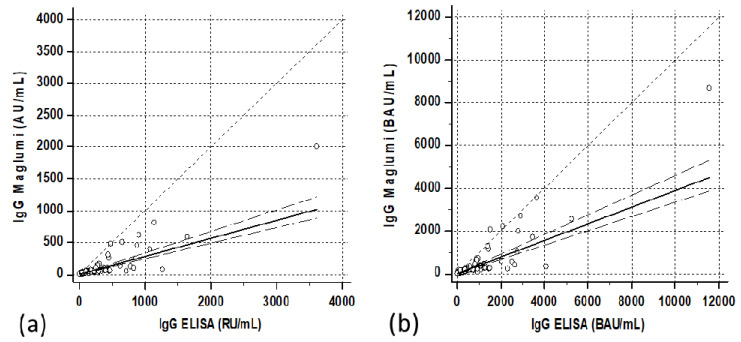
Correlations between ELISA (QuantiVac ELISA IgG, Euroimmun) and Maglumi (IgG anti-RBD, SNIBE) SARS-CoV-2 IgG levels in patients’ specimens. The trend lines represent the Passing–Bablock correlation. (**a**) Concentrations expressed in the respective units of each manufacturer [Maglumi= 0.581 (−0.21/2.64) ELISA + 0.284 (0.24/0.33)]. (**b**) Concentrations expressed as binding antibodies units (BAU) [Maglumi = 2.45 (−1.6/+10.6) +0.39 (0.33/0.46) ELISA].

The quantitative relationship showed a satisfactory correlation, although with a relatively scattered distribution of cases. Passing–Bablock regression resulted in “Maglumi= 0.284 (0.24/0.33) + 0.581 (−0.21/2.64) ELISA” (Figure 1a). In parenthesis were reported the confidence interval of intercepts and slopes.

The differences in the concentrations between the groups were statistically significant for both the methods (Kruskal–Wallis test *p* = 0.00002 for both Maglumi and ELISA, Table 2).

**Figure 2:**
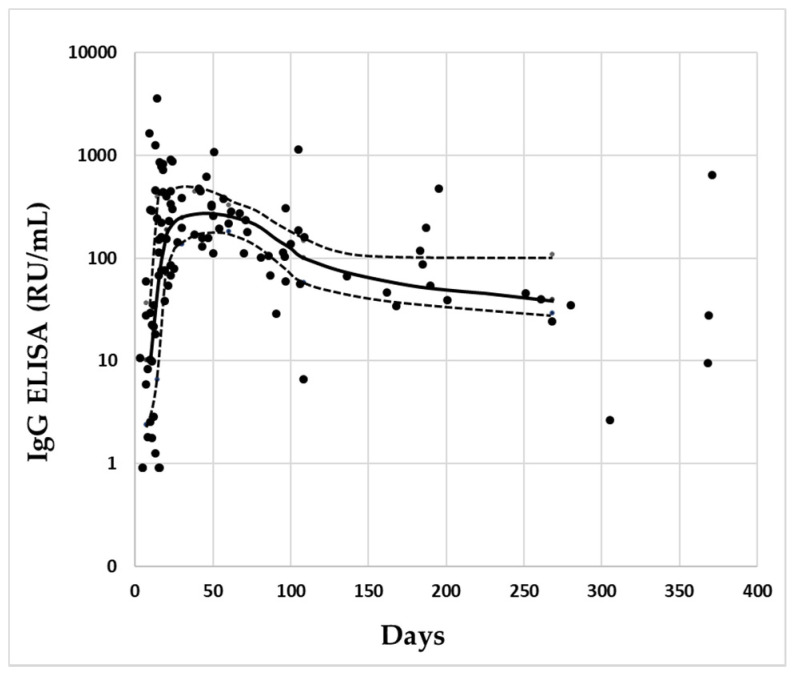
Distribution of IgG levels of the single specimens measured by ELISA in relation to the days since the onset of symptoms. In abscissa are reported the days from the onset of symptoms, in ordinate are reported the concentrations of IgG. The solid line connects the median concentrations of IgG for each class of cases, and the dotted line connects the respective 25–75° percentile. Black circles represent the singles specimens.

**Figure 3:**
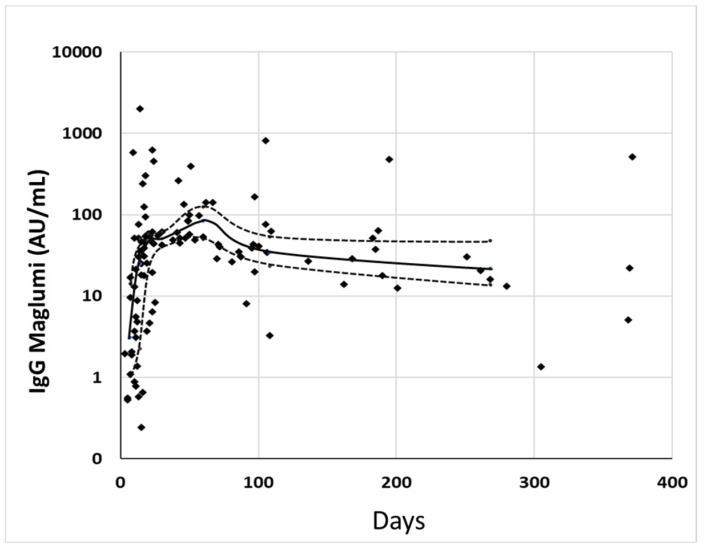
Distribution of IgG levels of the single specimens measured by Maglumi in relation to the days since the onset of symptoms. In abscissa are reported the days from the onset of symptoms, in ordinate are reported the concentrations of IgG. The solid line connects the median concentrations of IgG for each class of cases, and the dotted line connects the respective 25–75° percentile. Black circles represent the singles specimens.

The antibodies’ levels showed a similar time course with the two methods. After a rapid increase, the concentrations began to decrease slightly, after about 80–100 days (Figure 2 and Figure 3).

The ELISA method showed a sensitivity (calculated as the percentage of cases over the cut-off, with respect to the overall and clinically true positive cases of each group) of about 87% after 180 days, while the sensitivity of Maglumi remained at 100%.

**Figure 4:**
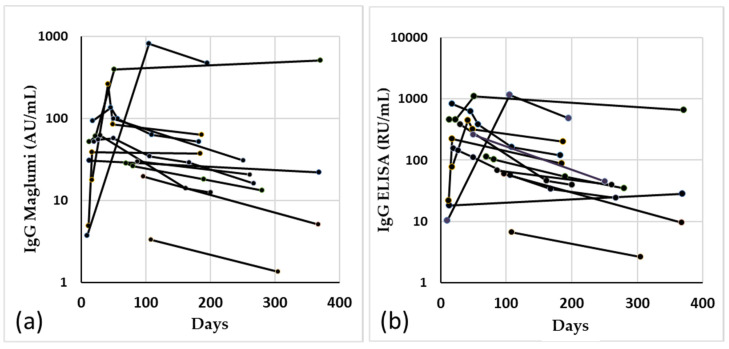
Spaghetti plot of the 13 patients with more than one withdrawal in more than 180 days from the onset of symptoms, measured by Maglumi (**a**) and ELISA (**b**).

The results of the determination with the two methods, in the 13 patients with more than one blood collection, at least up to 180 days after the onset of symptoms, are shown in Figure 4.

All the vaccinated subjects were positive 15 days after the first inoculum with the Maglumi method, while with the ELISA method, 4/22 cases had a negative result.

Fifteen days after the booster, all the samples were positive with both the methods, and the concentrations resulted in an amount that was more than 20 times larger than the first withdrawal (Table 3).

**Figure 5:**
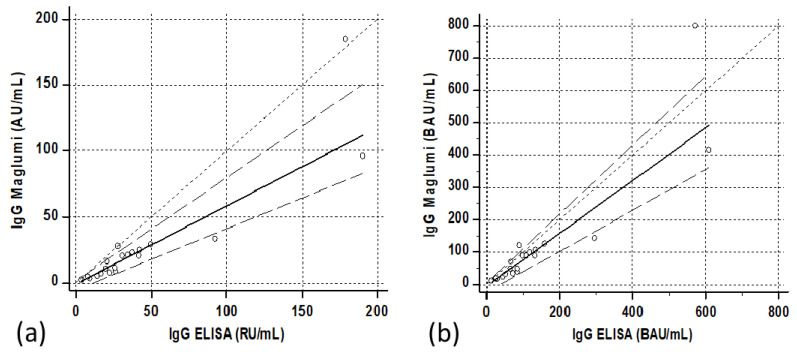
Correlation between ELISA and Maglumi methods in vaccinated subjects after the first dose. The trend lines represent the Passing–Bablock correlation. (**a**) Concentrations expressed in the respective units of each manufacturer [Maglumi= −0.89 (−6.1/+1.2) + 0.59 (0.47/0.78) ELISA. (**b**) Concentrations expressed as binding antibodies units [Maglumi = −5.1 (−26.8/+5.0) + 0.82 (0.64/1.07) ELISA]. Regression line (solid line), confidence interval lines (dashed lines) and identity line (dotted line) are displayed. Circles represent the single cases.

**Figure 6:**
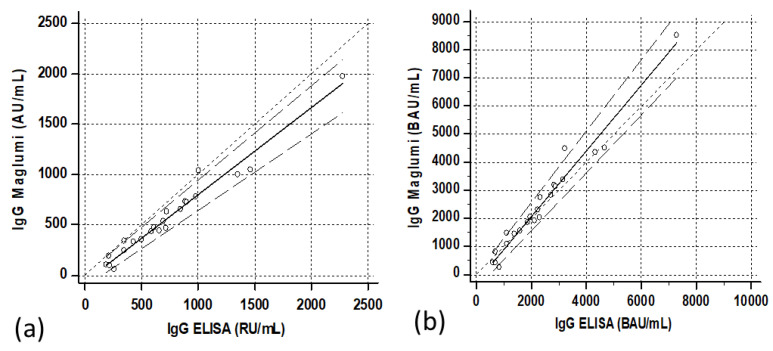
Correlation between ELISA and Maglumi methods in vaccinated subjects 15 days after the second dose. The trend lines represent the Passing–Bablock correlation. (**a**) Concentrations expressed in the respective units of each manufacturer [Maglumi= −52.4 (−107/+19.2) + 0.85 (0.74/0.92)]. (**b**) Concentrations expressed as binding antibodies units [Maglumi = −227.9 (−464/ + 69.9) + 1.14 (0.99/1.25) ELISA]. Regression line (solid line), confidence interval lines (dashed lines) and identity line (dotted line) are displayed. Circles represent the single cases.

The correlations between the two methods were satisfactory, especially after the second dose (Figure 5a and Figure 6a). The Passing–Bablock regressions were as follows: Maglumi = −0.89 (−6.1/+1.2) + 0.59 (0.47/0.78) ELISA for the specimens after the first dose, and Maglumi = −52.4 (−107/+19.2) + 0.85 (0.74/0.92) ELISA for the specimens after the second administration.

Three months after the second dose, the levels of antibodies drastically decreased (Table 3). The median of the percentage of the concentrations, compared to those found 15 days after the second dose, was 21% (10°–90° perc 11–33%) with Maglumi, and 24.4% (10°–90° perc 13–33%) with ELISA.

**Figure 7:**
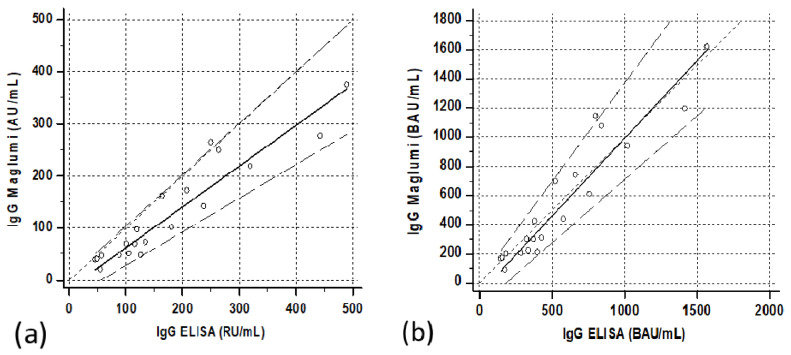
Correlation between ELISA and Maglumi methods in vaccinated subjects 3 months after the second dose. The trend lines represent the Passing–Bablock correlation. (**a**) Concentrations expressed in the respective units of each manufacturer [Maglumi= −16.2 (−37.5/+5.7) + 0.78 (0.65/0.98) ELISA]. (**b**) Concentrations expressed as binding antibodies units [Maglumi = −72.8 (−166.8/+24) + 1.07 (0.88/1.35) ELISA]. Regression line (solid line), confidence interval lines (dashed lines) and identity line (dotted line) are displayed. Circles represent the single cases.

The Passing–Bablock regression between the two methods was as follows: Maglumi = −16.2 (−37.5/+5.7) + 0.78 (0.65/0.98) ELISA (Figure 7a).

The correlations between the two methods after transformation into binding arbitrary units (BAU) resulted in Maglumi = 2.45 (−1.6/+10.6) + 0.39 (0.33/0.46) ELISA in patients, Maglumi = −5.1 (−26.8/+5.0) + 0.82 (0.64/1.07) ELISA in subjects vaccinated after the first dose and Maglumi = −227.9 (−464/+69.9) + 1.14 (0.99/1.25) ELISA in subjects vaccinated after the second dose. Three months after the second dose, the correlation was Maglumi = −72.8 (−166.8/+24) + 1.07 (0.88/1.35) ELISA (Figure 1b, Figure 5b, Figure 6b and Figure 7b).

## 4. Discussion

In this study, the performance of two assays, for the determination of antibodies against SARS-CoV-2 in patients, up to 10–12 months from the onset of symptoms, were compared. With both the methods, the antibodies concentrations tended to decline after a few months, but the levels persisted relatively high up to nearly a year after the symptoms (Table 2). Our data, especially those obtained with Maglumi, were roughly in accord with a model of the IgG anti-S decay in patients, which established a half-life of 229 days [17]. The positivity rates remained at 100% with the Maglumi method, while they dropped to 87% with the ELISA method.

In the vaccinated subjects, the presence of high concentrations of antibodies was already detectable after the first dose, but after the booster, they showed a significant increase, about 20 times compared to the first administration and, on average, 3 times the maximum concentrations reached by the patients after about two months from the onset of symptoms. These results are in agreement with previous findings for the method used [12,18,19]. However, in the vaccinated subjects the decrease in antibody concentrations was more rapid, given that 3 months after the second vaccination, they were reduced to less than a quarter. The correlations between the two methods have always been acceptable. However, in the patients the results were scattered and the ELISA method showed concentrations 3–4 times higher than those of Maglumi, while in the vaccinated subjects the concentrations between the two methods were closer and much better correlated. Moreover, the conversion of the results into BAU units improved the relationship between the two methods. However, only in the vaccinated subjects, there was no evidence of proportional error after the conversion, while in the patients, the difference between the two methods remained significant. The methods measured antibodies directed against the Spike 1 protein, but Maglumi determines antibodies against the receptor domain more specifically. This difference may partly justify the results in the patients. Considering that only the anti-Spike antibodies should be expressed in vaccinated patients, it could be speculated that the greater heterogeneity of the antibodies patterns in patients can cause the less close correlation between the two methods found in these subjects. The decrease in concentrations a few months after vaccination does not necessarily mean a reduction in protection. It is in fact possible that the protection is not directly proportional to the mere presence of antibodies, given the persistence of T-cell memory after infection [5]. In conclusion, both the methods had comparable behaviors, both in patients and in vaccinated subjects. In both cases, the antibody concentrations peaked and then decreased. However, in the vaccinated subjects, the peak reached a much higher level than in the patients, and the decrease was more rapid. Three months after the second injection, they showed concentrations comparable to those of patients after more than 6 months from the onset of the disease. A peculiar finding of the study was the failure of the conversion to binding antibodies units in harmonizing the two methods only in patients’ specimens. Further studies will be required to clarify this different behavior between patients and vaccinated subjects.

## Figures and Tables

**Table 1:** Characteristics of the studied patients.

Symptoms at the Onset of Disease	Frequency (%)
Fever	75.0
Cough	60.4
Dyspnea	29.2
Nausea	8.3
Asthenia	6.3
Others	10.5
**Disease severity**	***n* of patients**
Mild	9
Moderate	16
Severe	11
Critical	12
***n* of specimens**	***n* of patients**
1	19
2	12
3	7
4	7
5	2
6	1

The disease severity was classified according the WHO guidance “Laboratory testing for coronavirus disease (COVID-19) in suspected human cases”.

**Table 2:** Sensitivity and antibodies levels of ELISA (QuantiVac ELISA IgG, Euroimmun) and Maglumi (IgG anti-RBD, SNIBE) methods in the different patient’s specimens subdivided in time frames according to the day from the onset of symptoms.

		Positivity Rate		ELISA Levels (RU/mL)			Maglumi Levels (AU/mL)		
Days from Symptoms’ Onset	*n* of Specimens	ELISA	Maglumi	25 Percentile	Median	75 Percentile	25 Percentile	Median	75 Percentile
≤11 *	17	41.2%	76.5%	2.4	10.3	37.1	1.0	3.1	14.1
12–16 *	15	73.3%	80.0%	6.7	68.1	404.0	2.3	24.9	50.6
17–22	14	100.0%	100.0%	76.8	191.5	438.0	25.6	48.8	58.1
23–43	16	100.0%	100.0%	137.5	250.5	448.5	43.2	50.5	61.5
46–72	15	100.0%	100.0%	183.2	259.2	330.2	49.8	82.7	126.0
81–162 *	16	93.7%	100.0%	57.8	102.6	149.0	23.1	34.6	53.1
168–371 *	15	86.7%	100.0%	29.4	39.6	110.3	13.9	22.2	48.1

Asterisks represent the classes of cases significantly different from that with higher concentrations.

**Table 3:** Concentrations of antibodies anti SARS-CoV-2 in the vaccinated subjects measured by ELISA (QuantiVac ELISA IgG, Euroimmun) and Maglumi (IgG anti-RBD, SNIBE) methods, expressed both in the respective units of each manufacturer and in binding antibodies units.

		ELISA Levels		Maglumi Levels	
		Median	Interquartile Range	Median	Interquartile Range
Manufacturer-defined units/mL	15 days after the first dose	27.2	16.4–42	18.1	6.6–27.7
	15 days after the second dose	704.5	388.5–940	499	335.9–756.8
	3 months after the second dose	129.6	95.2–244	84.6	47.7–195
Binding antibody units/mL (WHO)	15 days after the first dose	87	58.1–113.8	78.4	30.8–113
	15 days after the second dose	2254	1243–3008	2160	1454–3277
	3 months after the second dose	366.1	206.7–844	414.7	305–781
